# Endophytic bacteria from in vitro culture of *Leucojum aestivum* L. a new source of galanthamine and elicitor of alkaloid biosynthesis

**DOI:** 10.1038/s41598-022-17992-5

**Published:** 2022-08-11

**Authors:** Agata Ptak, Emilia Morańska, Marzena Warchoł, Artur Gurgul, Edyta Skrzypek, Michał Dziurka, Dominique Laurain-Mattar, Rosella Spina, Anita Jaglarz, Magdalena Simlat

**Affiliations:** 1 Department of Plant Breeding, Physiology and Seed Science, University of Agriculture in Krakow, Łobzowska 24, 31-140 Krakow, Poland; 2 The Franciszek Górski Institute of Plant Physiology, Polish Academy of Sciences, Niezapominajek 21, 30-239 Krakow, Poland; 3 Centre for Experimental and Innovative Medicine, University of Agriculture in Krakow, Rędzina 1C, 30-248 Krakow, Poland; 4 INRAE, LAE, Université de Lorraine, 54000 Nancy, France; 5 Moredun Research Institute, Pentland Science Park, Bush Loan, Penicuik, EH26 0PZ UK

**Keywords:** Plant biotechnology, Bacteria, Biotechnology, Microbiology

## Abstract

*Leucojum aestivum* is known for its ability to biosynthesize alkaloids with therapeutic properties, among which galanthamine used for the treatment of Alzheimer's disease. New sources of this alkaloid are still being explored. In this study, a novel strain PLV of endophytic bacterium *Paenibacillus lautus* was isolated from in vitro *L*. *aestivum* plants. We report the whole genome sequence of that strain and its capacity to produce alkaloids and growth regulators. The effect of elicitation with autoclaved bacteria on the production of alkaloids was examined. Ten alkaloids were identified in bacteria extracts: galanthamine, lycorine, ismine, lycoramine, haemanthamine, tazettine, galanthine, homolycorine, 1,2-dihydrochlidanthine, and hippeastrine. The mean contents of galanthamine and lycorine were 37.51 µg/g of dry weight (DW) and 129.93 µg/g of DW, respectively. Moreover, isolated *P*. *lautus* strain synthesized: indole-3-acetic acid, t-zeatin, c-zeatin, kinetin, gibberellin A_1_, abscisic acid, salicylic acid, benzoic acid. In vitro elicitation of cultures with *P*. *lautus* increased dry biomass, stimulated galanthamine and lycorine production, contributed to 8,9-desmethylenebis (oxy)-7,9 dimethoxy-crinan biosynthesis, change pigments content, and antioxidant enzymes activities. Our findings for the first time point out that galanthamine can be synthesized by an microorganism. Moreover isolated strain can be used as a new elictor of Amaryllidaceae alkaloids biosynthesis.

## Introduction

*Leucojum aestivum* L. (summer snowflake) is a member of the Amaryllidaceae family. Plants belonging to this family are well known for their alkaloid contents. More than 650 alkaloids have thus far been identified in plants of this family and classified into groups according to a biochemical classification based on the biogenetic lineage and ring type. Georgiev et al.^1^ and Ka et al.^2^ performed detailed Amaryllidaceae alkaloid classifications. Amaryllidaceae alkaloids show a wide range of biological activities, and many of them have important pharmaceutical properties. Galanthamine is the first alkaloid to be used as a drug in the treatment of Alzheimer’s disease^3^. Amaryllidaceae alkaloids also exhibit anticancer activity, with lycorine exerting the strongest effect^4^. Clinical trials currently under way are exploring the use of lycorine in the treatment of cancer^5^. Recent studies have also shown that lycorine has the potential to combat SARS-CoV-2 infection due to its antiviral activity^6^.

The main methods of obtaining galanthamine are both chemical synthesis and extraction from plant materials. Many studies have also examined biotechnological methods using in vitro plant systems as alternatives to unprofitable production of galanthamine^1,7–10^. In vitro cultures of *L. aestivum* have been successfully established, and their ability to biosynthesize Amaryllidaceae alkaloids has been demonstrated. However, the amounts of alkaloids are not yet sufficiently large for mass production^11^. Therefore, it is important to explore new sources of galanthamine and the possibility of increasing alkaloid production.

It is well known that endophytes can produce the same or similar secondary metabolites as their host plants^12^. Medicinal plants have been shown to be great reservoirs of microorganisms, which can be valuable sources of specialized metabolites^13^. However, data on the isolation of endophytes from Amaryllidaceae plants and their ability to biosynthesize alkaloids are limited. Endophytic bacteria belonging to the genus *Bacillus* have been isolated from *L. aestivum* in vitro and in vivo bulbs^14^. Morare et al.^13^ identified five strains of endophytic bacteria belonging to the genera *Staphylococcus*, *Bacillus*, and *Acinetobacter* in *Crinum macowanii* bulbs. While Liu et al.^15^ and Zhou et al.^16^ isolated 188 bacterial endophytes and 108 fungal strains from *Lycoris radiata*. In turn Wang et al.^17^ isolated endophytic fungi from *Narcissus tazetta*. Endophytic bacteria of *L. aestivum* biosynthesized five Amaryllidaceae alkaloids^14^. While endophytic bacteria derived from *C. macowanii* biosynthesized three alkaloids^18^. However, no endophytes capable of synthesizing galanthamine have been reported in the literature.

Endophytic microorganisms can also produce growth regulators, thus promoting plant growth and development even under unfavourable abiotic and biotic stresses^19–21^. However, the ability of endophytic microorganisms isolated from Amaryllidaceae plants to biosynthesize growth regulators has not yet been studied, although some endophytes have been shown to stimulate *L. radiata* growth^15^.

Apart from being valuable sources of metabolites, endophytes can also be used as elicitors of metabolite biosynthesis in plants. Important biotic elicitors include raw or autoclaved endophytic bacterial cells^22,23^. The action of such factors depends on many variables, such as the plant genotype, the type of in vitro culture, and the type of bacteria. Co-cultures with endophytic *Bacillus altitudinis* have been shown to increase ginsenoside production in *Panax ginseng* root cultures, while inoculation of *Withania somnifera* plants with *Pseudomonas* spp. stimulated withanolide biosynthesis^24,25^. Moreover, autoclaved endophytic *Bacillus megaterium* has been reported to stimulate quinone-methide triterpene production in adventitious *Peritassa campestris* root cultures^26^*.* To our knowledge, no biotic elicitors have been studied in in vitro *L. aestivum* cultures.

The primary objective of this study was isolation, identification, and whole genome sequence analysis of bacteria from in vitro plants of *L. aestivum*. A second aim was to investigate the ability of the identified bacteria to biosynthesize Amaryllidaceae alkaloids, especially galanthamine. Next, we checked the capacity of endophytic bacteria to biosynthesize growth regulators. Finally, the study aimed to explore the potential of autoclaved endophytic bacteria as new elicitors of in vitro *L. aestivum* plant growth in a RITA^®^ bioreactor with a view to increase plant biomass and Amaryllidaceae alkaloid production.

## Results and discussion

### Isolation and molecular identification of endophytic bacteria from in vitro *L. aestivum plants*

Endophytic bacteria are often observed in in vitro plant cultures. They are known to live inside plant tissues without the plants exhibiting symptoms of disease^27^. In this study, no significant outgrowth of bacteria was observed during micropropagation of *L. aestivum* plants (see Supplementary Fig. S1a). The only indication of the presence of bacteria was a slight smear around the bulbs or roots. However, increased bacterial growth was noted after transferring the *L*. *aestivum* culture from 5 to 25 °C to the medium containing melatonin. The endophytic bacterium described in this study was isolated from leaf and bulbs fragments of in vitro-grown *L. aestivum* and cultured on solidified lysogeny broth medium (LB)^28^ (see Supplementary Fig. S1b, c). Bacterial colonies were classified as Gram positive and further identified by whole genome sequence analysis. Genome sequencing of the isolated strain resulted in 12,903,512 paired reads of which 12,102,198 (93%) passed initial filtering. Those reads generated in total 3,6 Gb of sequence which covered *Paenibacillus lautus* genome 496 times on average. The total estimated genome size of isolated strain was 7,809,730 bp. Assembly and scaffolding of the reads resulted in 61 scaffolds coming form 109 cotigs with N50 of 376,288 and L50 of 7. Quality assessment for *Paenibacillus lautus* PLV strain assembly is presented in Supplementary Table S1. The ungapped total plasmid length was estimated at 199,621 bp.

Annotation of the assembled sequence allowed identification of 7,154 genes, of which 6,917 were protein coding genes. The genes also comprised 94 RNA genes, which included: 18 rRNA genes, (5S, 16S, 23S); 72 tRNAs and four ncRNAs. Assemblies were finally deposited in the GenBank under the Bio Project number PRJNA751742 and the strain was labeled as PLV.

**Figure 1:**
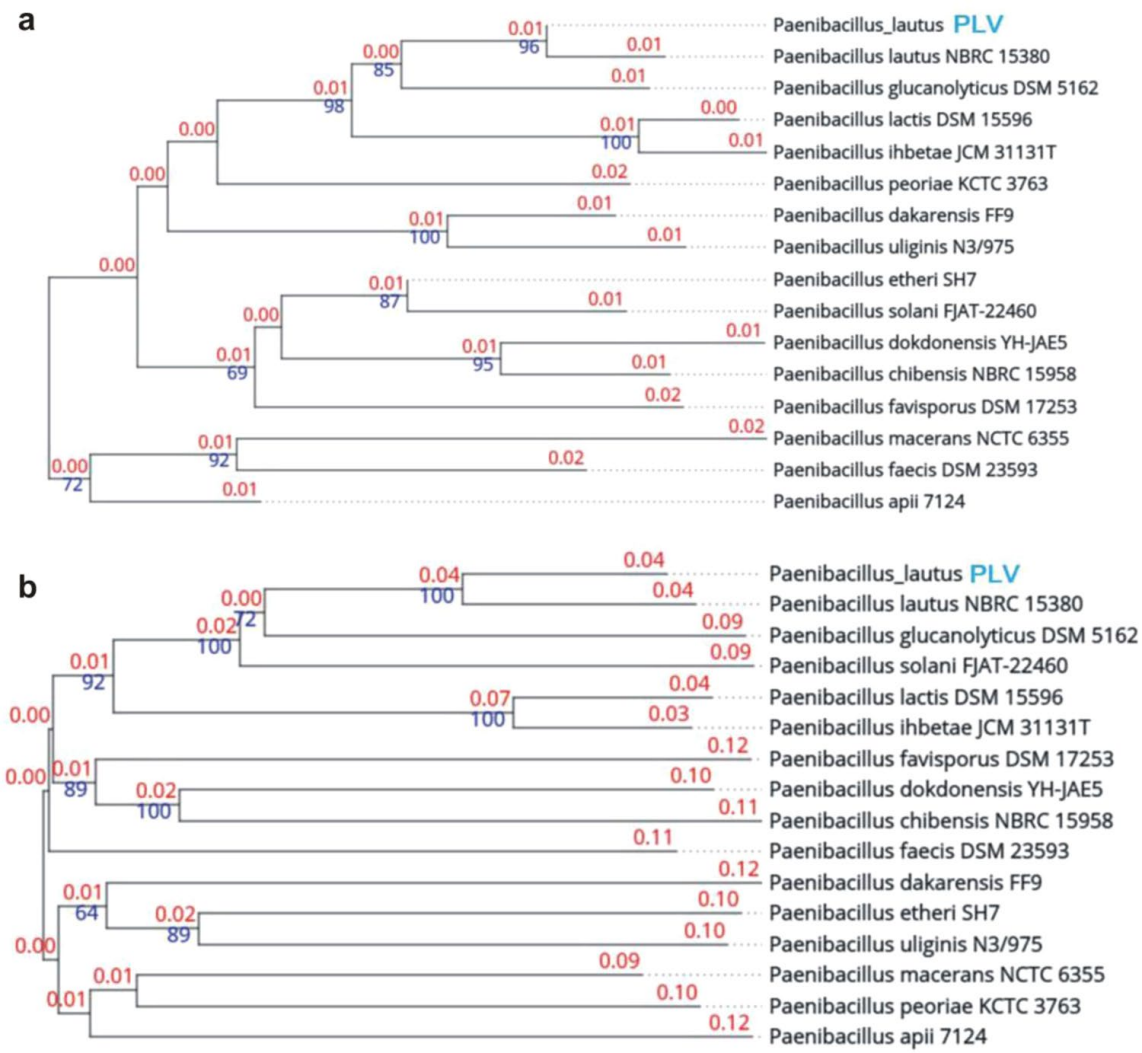
(**a**) SSU tree. Tree inferred with FastME 2.1.6.1^29^ from GBDP distances calculated from 16S rDNA gene sequences. The branch lengths are scaled in terms of GBDP distance formula d5. The numbers above branches are GBDP pseudo-bootstrap support values > 60% from 100 replications, with an average branch support of 76.9%. The tree was rooted at the midpoint^30^. (**b**) Genome tree—Tree inferred with FastME 2.1.6.1^29^ from GBDP distances calculated from genome sequences. The branch lengths are scaled in terms of GBDP distance formula d5. The numbers above branches are GBDP pseudo-bootstrap support values > 60% from 100 replications, with an average branch support of 75%. The tree was rooted at the midpoint^29^.

Based on TYGS analysis we found that (when considering 16S rDNA sequence) the most related species/strains were: *Paenibacillus lautus* (NBRC15380), *Paenibacillus glucanolyticus* (DSM5162), *Paenibacillus lactis* (DSM15596) and *Paenibacillus ihbetae* (JCM 31131T). Similar results were obtained when whole-genome tree was analyzed, however, *Paenibacillus ihbetae* was replaced by *Paenibacillus solani* (FJAT-22460) in the cluster of the most similar species (Fig. 1). To our knowledge, this is the first study to report the isolation of *P*. *lautus* from in vitro *L. aestivum* plants. Previously, Spina et al.^14^ isolated bacteria belonging to the genus *Bacillus* from in vivo and in vitro *L. aestivum* bulbs.

### Ability of *P. lautus* PLV strain to biosynthesize Amaryllidaceae alkaloids and plant growth regulators

**Figure 2:**
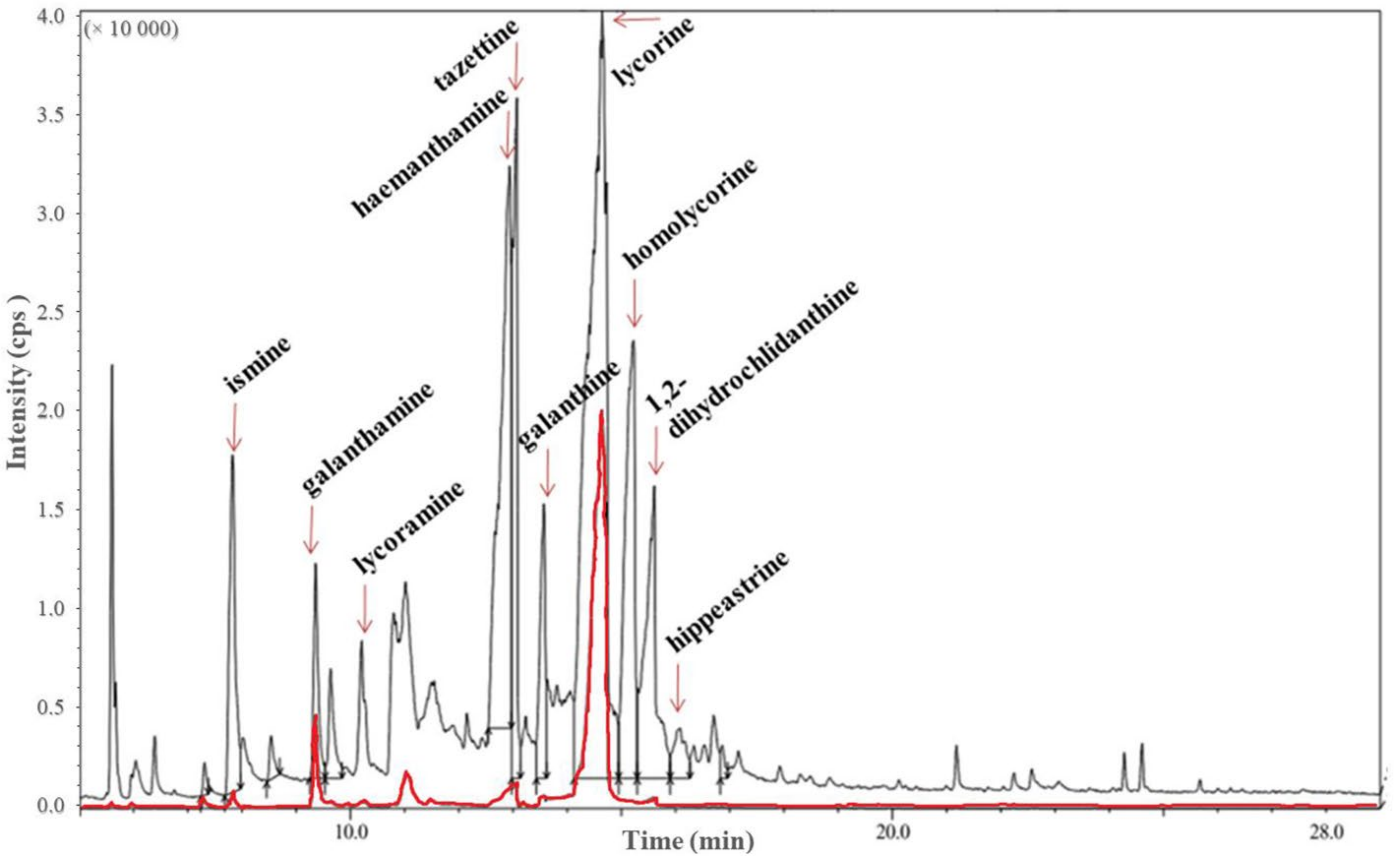
GC–MS total ion current chromatograms of the alkaloid fraction from *Paenibacillus lautus* strain PLV extract (upper trace) and alkaloids standard mixture (lower, red-shaded trace), acquired in scan mode. Identification based on library search, details given in Table S2.

We demonstrated that endophytic *P. lautus* PLV strain isolated from in vitro *L. aestivum* plants synthesizes Amaryllidaceae alkaloids. To investigate the chemical composition of purified extracts, a gas chromatography–mass spectrometry (GC–MS) was used and ten alkaloids were identified (Fig. 2) by comparing the measured data with previously published data^14^ and using the NIST library^31^. A detailed list of the identified alkaloids is presented in Supplementary Table S2. It is noteworthy that two of the most important alkaloids synthesized by *P. lautus* PLV strain were identified by comparison with authentic compounds and quantified by liquid chromatography–mass spectrometry (LC–MS): galanthamine (37.51 µg/g of DW) and lycorine (129.93 µg/g of DW) (Table 1). The accumulation of galanthamine and lycorine by isolated bacteria is comparable to or higher than that in *L. aestivum* plant, shoot, and callus cultures^1,32^. Earlier reports showed the possibility of biosynthesis: lycorine, tazettine, pseudolycorine, acetylpseudolycorine, and 1,2-dihydro-chlidanthine by the endophytic bacteria of *L. aestivum* and lycorine, crinamidine, and powelline by the bacteria from *C. macowanii*^14,18^. It is worth emphasizing, that this is the first information about the synthesis of galanthamine by endophytic microorganisms. It is noteworthy that *P. lautus* PLV strain produced 10 Amaryllidaceae alkaloids. By comparison, in vitro *L. aestivum* plants most often synthesize two to seven alkaloids^32,33^. Moreover, alkaloids such as galanthine and hippeastrine identified in *P. lautus* PLV strain extracts have not previously been isolated from *L. aestivum* cultures. Galanthine is an alkaloid characteristic of *Galanthus woronowii*^34^, among others, while hippeastrine is typical of *Hippeastrum* sp.^35^. It is worth noting that hippeastrine has a strong antiproliferative effect and may find potential applications in the treatment of cancer^36^.

**Table 1:** Amaryllidaceae alkaloids and growth regulators content in *Paenibacillus lautus* strain PLV.

Alkaloids	Content [µg/g of DW] ± SD
Galanthamine	37.51 ± 1.5
Lycorine	129.93 ± 3.58

Growth regulators*	Content [ng/g of DW] ± SD
t-Z	5.4 ± 0.49
c-Z	3.5 ± 0.51
K	146.4 ± 32.39
BeA	6964.1 ± 895.45
IAA	341.5 ± 128.67
SA	771.7 ± 95.30
GA_1_	14.3 ± 0.47
ABA	181.4 ± 30.14
JA	1991.7 ± 84.39

The results are means of three replicates (n = 3) ± SD. Different letters indicate a significance difference at *P* < *0.05* according to ANOVA and Duncan’s test.

DW, dry weight.

*Z, zeatin; K, kinetin; BeA, benzoic acid; IAA, indoleacetic acid; SA, salicylic acid; GA_1_, gibberellin A_1_; ABA, abscisic acid; JA, jasmonic acid.

**Figure 3:**
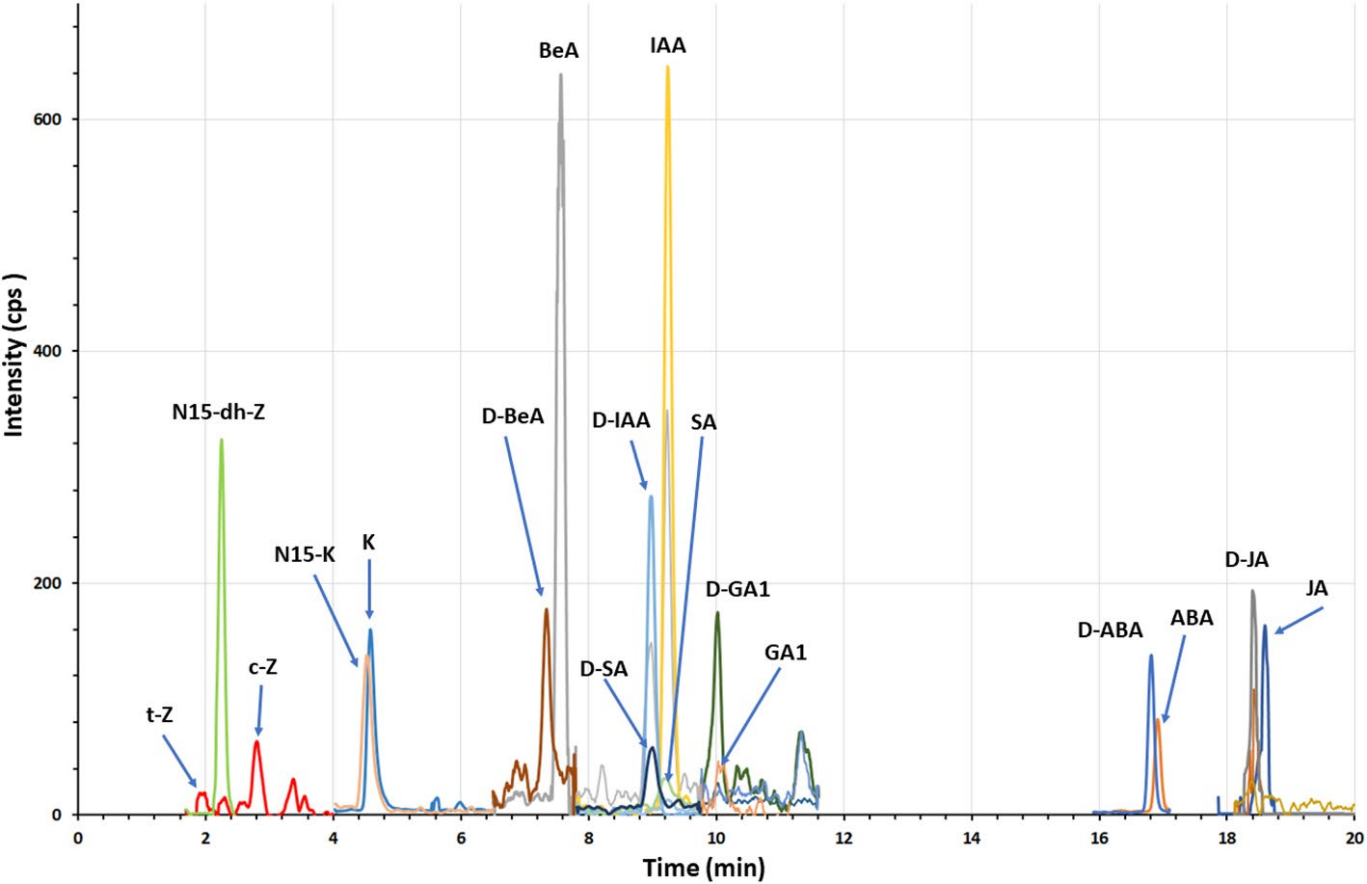
UHPLC-MS/MS chromatogram of multiple reaction monitoring (MRM) transitions for the analysed plant growth regulators (zeatin: Z, kinetin: K, benzoic acid: BeA, indoleacetic acid: IAA, salicylic acid: SA, gibberellin A1: GA_1_, abscisic acid: ABA, and jasmonic acid: JA). Details given in Table S3.

Chromatographic analyses showed that *P. lautus* strain PLV isolated from in vitro cultures of *L. aestivum* is capable of biosynthesizing auxin (indole-3-acetic acid: IAA), cytokinins (t-zeatin, c-zeatin: Z, and kinetin: K), gibberellin A_1_ (GA_1_), abscisic acid (ABA), jasmonic acid (JA), salicylic acid (SA), and benzoic acid (BeA) (Fig. 3, Supplementary Table S3). It is worth noting that the endophytic bacteria synthesized the greatest amount of stress-related hormones, such as BeA, JA and SA (6964.1, 1991.7, 771.7 ng/g DW, respectively). A slightly lower but still high amount was also reported for ABA (181.4 ng/g DW) (Table 1). It is known that endophytic bacteria can protect their host plants from phytopathogens induced systemic resistance (ISR), therefore endophytes very often synthesize these hormones. Endophytic bacteria can initiate ISR using among others SA and JA^37^. Endogenous and exogenous jasmonates and SA regulated also the expression of the genes related to biosynthesis of secondary metabolites^38^. Perhaps the biosynthesis of Amaryllidaceae alkaloids by endophytic *P. lautus* is associated with a high content of endogenous stress hormones. Research has shown that exogenous methyl jasmonate and SA stimulated galanthamine and lycorine biosynthesis in in vitro culture of *L*. *aestivum*^39^. To our knowledge, this is the first report on the biosynthesis of growth regulators by *P. lautus* isolated from in vitro cultures of *L. aestivum*. No such studies have been conducted on other endophytic bacteria isolated from plants of the Amaryllidaceae family. While the production of growth regulators by endophytic bacteria has been reported in the literature, for example IAA was produced by *Paenibacillus polymyxa* isolated from *Lilium lancifolium* bulbs and *Triticum aestivum* roots^40,41^. Previous studies have also reported the synthesis of cytokinins by endophytic *Pseudomonas resinovorans* and *P. polymyxa* derived from *Gynura procumbens* leaves and *Bacillus*, *Micrococcus*, *Pseudomonas*, *Flavobacterium*, and *Serratia* spp. from tropical legume plants^40,41^. Endophytic *Bacillus* sp. isolated from *Clerodendrum colebrookianum* plants produced kinetin and 6-benzyladenine^42^, *Bacillus amyloliquefaciens*, isolated from *Oryza sativa* seeds gibberellins^42^, and *Bacillus pumilus* from *Helianthus annuus* JA^37^. In turn *Pseudomonas tremae* and *Curtobacterium herbarum* isolated from *Salix babylonica* synthesize SA^43^.

### Effect of *P. lautus* strain PLV elicitation on dry weight increment in *L. aestivum* in vitro plants

Elicitors can induce stress responses in in vitro cultures and cause morphological, physiological, biochemical, and molecular changes, thereby influencing growth. Thus, the biomasses of plant cultures may decrease after treatment with elicitors^23^. This phenomenon was not observed in our research. The dry weight (DW) of *L. aestivum* control and elicited with autoclaved bacteria plants increased throughout the culture period (Table 2). The highest increase in dry biomass (0.53 g) was achieved on day 28 with the 0.04% elicitor. This increase was about 1.3 times higher than that observed in the control plants on day 28. However, on day 7, the greatest increases in dry biomass were observed in the control plants and in the plants treated with the 0.01% elicitor. On day 14, the 0.02% elicitor stimulated dry biomass accumulation in *L. aestivum* plants. These increases were approximately 1.89 times lower than those observed in plants treated with the 0.04% elicitor for 28 days.

**Table 2:** Effect of *Paenibacillus lautus* strain PLV elicitation on dry biomass increments and pigments content in *L. aestivum* in vitro plants.

Bacterial extract [%]	Time [days]	DW increment [g]	Pigments content [µg/g FW]		
			Chlorophyll *a*	Chlorophyll *b*	Carotenoids
0	7	0.25 ± 0.04 cde	22.21 ± 7.06 b	16.57 ± 5.36 b	3.90 ± 1.90 c
0.01	7	0.26 ± 0.06 cd	37.08 ± 6.39 a	23.96 ± 2.08 ab	9.15 ± 0.55 b
0.02	7	0.20 ± 0.01 f.	26.44 ± 1.53 b	19.04 ± 1.19 b	5.79 ± 1.18 bc
0.04	7	0.21 ± 0.01 ef	23.16 ± 2.08 b	18.72 ± 0.25 b	7.98 ± 2.57 bc
0	14	0.18 ± 0.01 f.	26.12 ± 7.85 b	20.92 ± 4.69 b	6.65 ± 3.33 bc
0.01	14	0.23 ± 0.01 def	23.58 ± 2.47 b	18.74 ± 1.31 b	5.34 ± 0.2 bc
0.02	14	0.28 ± 0.03 c	20.67 ± 2.39 b	19.28 ± 3.69 b	6.49 ± 2.23 bc
0.04	14	0.14 ± 0.01 g	27.28 ± 1.42 b	19.09 ± 2.93 b	6.82 ± 1.74 bc
0	28	0.41 ± 0.03 b	29.72 ± 2.93 ab	22.99 ± 0.95 ab	7.80 ± 2.67 bc
0.01	28	0.39 ± 0.01 b	26.11 ± 4.77 b	25.77 ± 2.46 a	15.55 ± 2.18 a
0.02	28	0.43 ± 0.03 b	20.05 ± 2.81 b	21.82 ± 0.39 b	8.60 ± 0.05 b
0.04	28	0.53 ± 0.02 a	23.20 ± 2.70 b	23.81 ± 2.17 ab	7.88 ± 2.62 bc

The results are means of three replicates (n = 3) ± SD. Different letters indicate a significance difference at *P* < *0.05* according to ANOVA and Duncan’s test; DW: dry weight, FW: fresh weight.

Thus, the stimulating effect of the elicitor could be related to the ability of *P. lautus* strain PLV to produce growth regulators. Endogenous growth regulators, along with exogenous ones, play a key role in in vitro morphogenesis. The most important are auxins and cytokinins. In this study, *P. lautus* strain PLV biosynthesized auxin (IAA) and cytokinins (t-zeatin, c-zeatin, and kinetin) (Table 1). There is little information regarding the elicitation of tissue cultures with autoclaved endophytic bacteria. Song et al.^24^ observed a stimulating effect of live endophytic bacteria on the growth of adventitious *P. ginseng* roots.

### Effect of *P. lautus* strain PLV elicitation on pigments content and antioxidant enzymes activity

The analysis has proven the influence of bacterial elicitor on the production of pigments in extracts from *L. aestivum* plants. Elicitation of *L. aestivum* cultures with the 0.01% elicitor for seven days increased the chlorophyll *a* content about 1.6 times in comparison with control and other treated plants (Table 2). The highest contents of chlorophyll *b* (25.77 µg/g of fresh weight: FW) and carotenoids (15.55 µg/g of FW) were also recorded with this concentration, but on day 28 (Table 2). Previous studies have investigated the effects of endophyte inoculation on the amounts of chlorophyll *a* in plants. For example, inoculation of *Papaver somniferum* cv. Sampada plants with a *Bacillus* sp. increased the chlorophyll content^44^. Root-associated endophytic fungi enhanced the chlorophyll content, photosynthesis rate, stomatal conductance, and transpiration rate of *Cucumis sativus* plants^45^. Inoculation of *W*. *somnifera* plants with live fungal endophytes improved their photosynthetic efficiency. The treated plants had higher chlorophyll and carotenoid amounts than control plants^25^.

**Figure 4:**
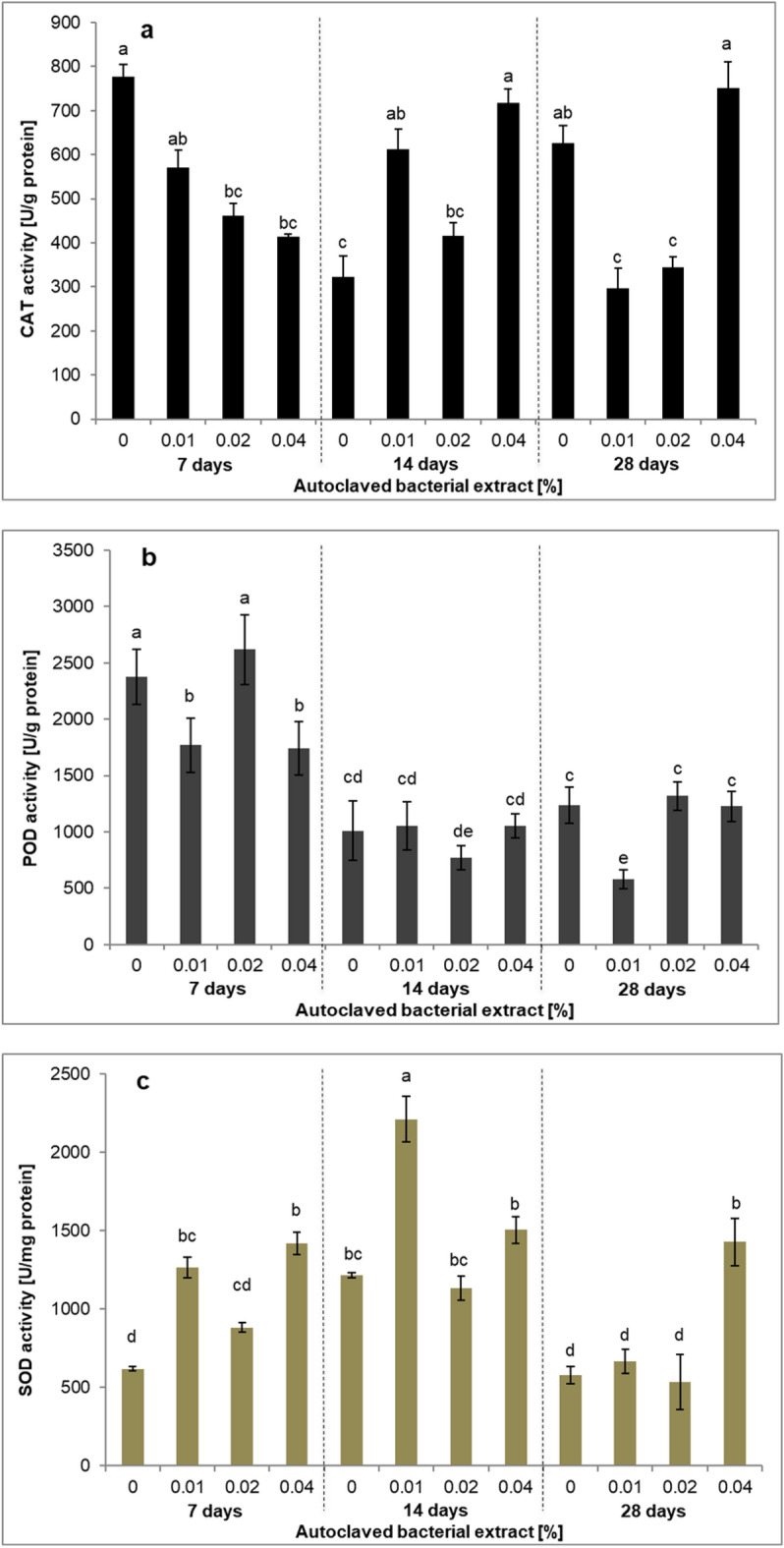
Effect of *Paenibacillus lautus* strain PLV elicitation on (**a**) CAT (catalase), (**b**) POD (peroxidase) and (**c**) SOD (superoxide dismutase) activity in *L. aestivum* in vitro plants. The results are means of three replicates (n = 3). Error bars represent ± SD. Different letters indicate a significance difference at *P* < 0.05 according to ANOVA and Duncan’s test.

Studies have shown that when plants are exposed to stressful conditions, their antioxidant systems play key protective roles by increasing the activity of antioxidant enzymes^29^. It is also known that antioxidant enzyme activity can results enhancement biosynthesis of secondary metabolites. Treatment with elicitors induces oxidative stress. In this study, the activities of the antioxidant enzymes catalase (CAT), peroxidase (POD), and superoxide dismutase (SOD) were examined (Fig. 4). The highest CAT activity were recorded in control plants on day 7 and in plants treated with the 0.04% elicitor on days 14 and 28 (Fig. 4a). The highest POD activity was noted on day 7 in control plants and in plants treated with the 0.02% elicitor. Slightly lower activity, but higher than under other conditions, was observed with the 0.01% and 0.04% elicitors on day 7 (Fig. 4b). This high POD activity on day 7 may have been caused by stress induced when the plants were transferred from the solid medium to the RITA^®^ bioreactor and were thus forced to gradually adapt to new conditions. The highest SOD activity was recorded on day 14 with the 0.01% elicitor. It is worth noting that, regardless of the conditions, SOD reached higher values on day 14 (average 1.515 U/µg of protein) than on days 7 (average 1.06 U/µg of protein) and 28 (average 0.8 U/µg of protein) (Fig. 4c). These results indicate that in *L. aestivum* cultures, the activity of antioxidant enzymes depends not only on the concentration of the bacterial elicitor but also on the duration of its action. In the case of *L. aestivum* cultures, the influence of abiotic elicitors, such as melatonin and sugars, on the activity of antioxidant enzymes has been studied. However these analyses were performed only on the 28th day of culture^10,32^.

### Effect of *P. lautus* strain PLV elicitation on the biosynthesis of Amaryllidaceae alkaloids in in vitro* L. aestivum* plants

The qualitative analysis of alkaloids from *L. aestivum* plants was performed using GC–MS. The analyses showed various Amaryllidaceae alkaloids in complex alkaloid fractions in plant materials. From one (lycorine) to four alkaloids (galanthamine, crinan-3-ol, demethylmaritidine and lycorine) were detected (Supplementary Fig. S2, Table 3). Alkaloid biosynthesis varied according to the in vitro conditions and the culture duration (Table 3). On day 7, the 0.01% and 0.02% elicitors stimulated the biosynthesis of two alkaloids: crinan-3-ol and lycorine. By day 28, the biosynthesis of demethylmaritidine was observed both in control and in the presence of 0.01% and 0.02% elicitors while 0.04% elicitor stimulated the biosynthesis of 8,9-desmethylenebis (oxy)-7,9 dimethoxy-crinan which was not observed in any plant materials. This was consistent with our earlier observation that the use of elicitors in a culture medium stimulated the biosynthesis of alkaloids that are usually not present in in vitro *L. aestivum* cultures^32^. The highest number of alkaloids was observed in control plants on day 28 (galanthamine, crinan-3-ol, demethylmaritidine and lycorine). It is worth emphasizing that on the 28th day of the culture the highest activity of CAT and SOD was observed (Fig. 4).

**Table 3:** Amaryllidaceae alkaloids identified by GC–MS (% of TIC) in *L. aestivum* in vitro plants during feeding with various concentrations of *Paenibacillus lautus* strain PLV (0%, 0.01%, 0.02% and 0.04%).

Alkaloid	Formula	Base peak	Retention time [min]	Retention index	0%	0.01%	0.02%	0.04%	0%	0.01%	0.02%	0.04%	0%	0.01%	0.02%	0.04%
					7 days				14 days				28 days			
Galanthamine	C_17_H_21_NO_3_	287	9.30	2213	nd	nd	nd	36.7%	nd	nd	nd	nd	6.1%	nd	nd	8.5%
Crinan-3-ol (elwesine)	C_16_H_19_NO_3_	272	9.86	2164	–	47%	34.9%	–	–	–	–	–	11.6%	–	–	–
8,9-Desmethylenebis (oxy)-7,9 dimethoxy-crinan	C_17_H_23_NO_2_	273	10.74	2010	–	–	–	–	–	–	–	–	–	–	–	15.8%
Demethylmaritidine	C_16_H_19_NO_3_	273	10.74	221	–	–	–	–	–	–	–	–	20.2%	15.7%	16.1%	–
Lycorine	C_16_H_17_NO_4_	226	14.03	2263	79.2%	34.2%	41%	28.7%	74.8%	67.3%	90.8%	66.5%	54.5%	74.1%	71.9%	65.4%

TIC, Total Ion Chromatogram; nd, not detected; (−), no alkaloid

**Figure 5:**
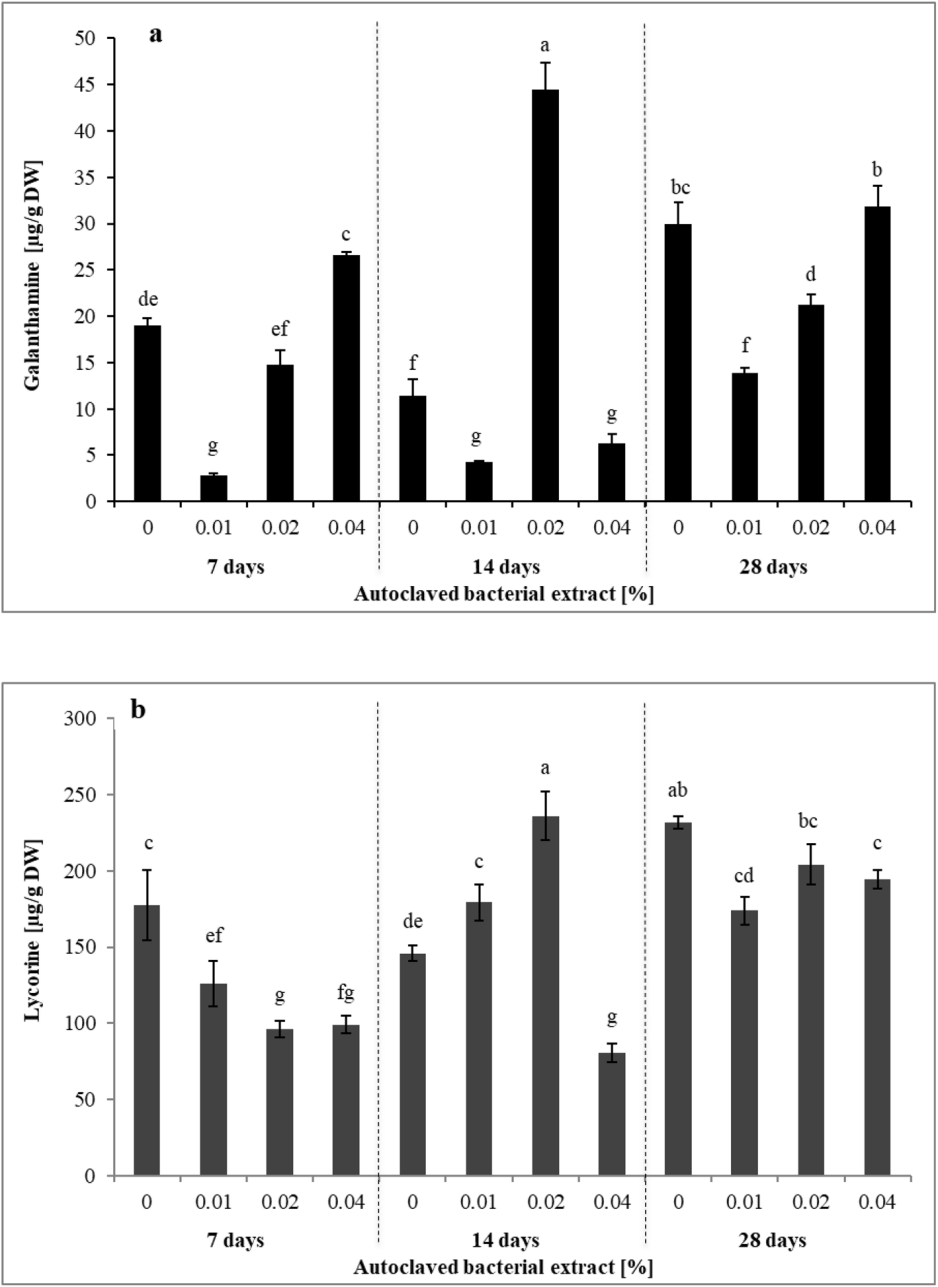
Effect of *Paenibacillus lautus* strain PLV elicitation on (**a**) galanthamine, (**b**) lycorine content in *L. aestivum* in vitro plants. The results are means of three replicates (n = 3) of analysis using LC–MS. Error bars represent ± SD. Different letters indicate a significance difference at *P* < *0.05* according to ANOVA and Duncan’s test. DW: dry weight.

Autoclaved endophytic *P. lautus* strain PLV were found to increase the production of galanthamine and lycorine in *L. aestivum* tissue cultures (Fig. 5). It is worth noting that LC–MS used for the quantification of alkaloids from plants allowed the detection of galanthamine and lycorine in all the samples studied. The highest galanthamine content (44.47 µg/g of DW) was observed in plants treated with the 0.02% elicitor for 14 days. This content was 3.9 times higher than that in control plants that grew for 14 days (Fig. 5a). The 0.02% elicitor also stimulated lycorine biosynthesis. On day 14, a content of 235.73 µg/g of DW was recorded, compared to 145.75 µg/g of DW in control plants (Fig. 5b). These findings confirm the results of a previous study reporting that the optimum times and concentrations of abiotic elicitor treatments are important for the production of Amaryllidaceae alkaloids^39^. However, there is no information in the literature regarding the effects of autoclaved endophytic bacterial elicitation on Amaryllidaceae alkaloid biosynthesis. It should also be noted that on day 14, the 0.02% elicitor also increased the DW biomass of *L. aestivum* plants (Table 2). The large increase in biomass combined with the high production of alkaloids are very important for industrial production. Liu et al.^15^ showed that inoculation of in planta *L*. *radiata* with live endophytic bacteria increased the plant DW, resulting in an increase in the total yield of alkaloids. There is also little information on the use of endophytic bacteria in the biosynthesis of specialized metabolites in in vitro cultures, as endophytic fungi have typically been used for this purpose^16^. Inacio et al.^26^ found that eliciting *P*. *campestris* root cultures with autoclaved endophytic *B. megaterium* stimulated the biosynthesis of 22 β-hydroxymaitenin. The use of live bacteria to stimulate the biosynthesis of specialized metabolites in in vitro cultures has also been reported. For example, a co-culture with endophytic bacteria increased ginsenoside production in *P*. *ginseng* root cultures^24^. Also, *Pseudomonas* and *Streptomyces* spp. stimulated withaferin-A, 12-deoxywithstramonolide, and withanolide A production in in vitro *W*. *somnifera* plants^25^.

## Conclusion

This research was conducted in an attempt to find a new producer of industrially valuable Amaryllidaceae alkaloids and a way to increase their biosynthesis in in vitro cultures of *Leucojum aestivum*. To the best of our knowledge, this is the first report on the possibility of galanthamine biosynthesis by an endophytic microorganism. *Paenobacillus lautus* strain PLV, isolated for the first time from *L. aestivum* in vitro plants showed great potential for the synthesis also others Amaryllidaceae alkaloids. Moreover, *P. lautus* strain PLV biosynthesized nine growth regulators and stimulated in vitro plant growth. Furthermore, it may elicit the biosynthesis of alkaloids that are not observed in untreated plants. The obtained results provide a new perspective on Amaryllidaceae alkaloids production, especially galanthamine. It will also be helpful in research on understanding the still unexplored mechanisms of galanthamine biosynthesis in ‘plant’ and ‘bacterial’ systems and the ‘plant-bacterial’ relationship.

Future research should focus on larger-scale biosynthesis of Amaryllidaceae alkaloids by *P. lautus* strain PLV using a fermenter, as well as on the optimization of elicitation in *L. aestivum* cultures.

## Materials and methods

### Isolation of endophytic bacteria from *L. aestivum* plants

*L. aestivum* plants L. obtained from leaves explants isolated from bulbs purchased from a local Polish market, via somatic embryogenesis were maintained in vitro for two years using periodic (eight week) subcultures with a fresh Murashige and Skoog (MS) medium^46^ containing 60 g/L of sucrose. The pH of medium was adjusted to 5.8, and the cultures were maintained at 5 °C in darkness. Then, the plants were transferred to a 25 °C environment with an MS medium supplemented with 10 µM of melatonin according to Ptak et al.^10^. Afterwards, plant leaves and bulbs with visible bacteria were dissected into small pieces and transferred to a solid lysogeny broth medium (LB)^28^ and incubated at 37 °C for 72 h. To obtain single colonies, the bacteria were transferred to a fresh LB medium via streaking (using a platinum loop) and incubated at 37 °C for 24 h. The authors confirm that all methods used were performed in accordance with the relevant guidelines and legislation.

### Molecular identification of endophytic bacteria

Bacteria obtained from LB plates were stained with standard Gram technique for microscopic observation and to determine the DNA extraction method. A single bacterial colony was used to inoculate 10 mL of LB medium. After a 24 h incubation (37 °C on a shaker at 100 rpm) the cells were harvested by centrifugation at 6000 xg, for 10 min (Biofuge Stratos, Heraeus, Germany). Pellet was used for genomic DNA extraction using Genomic Maxi AX kit (A&A Biotechnology, Poland) according to manufacturer protocol with pre-treatment for Gram-positive bacteria including additional digestion steps with lysozyme and proteinase K. DNA concentration was assessed with NanoDrop2000c (Thermo Fisher Scientific, USA). The obtained genomic DNA of the endophytic bacteria was used for WGS (whole genome sequencing). The sequencing library was prepared commercially using Illumina’s Nextera XT DNA library kit, and quality was assessed by Qubit DNA Assay (Thermo Fisher; Waltham, Massachusetts, USA), Agilent Bioanalyzer 2100 (Agilent; Santa Clara, CA, USA), and qPCR at Applied Biological Materials Inc. (Richmond, BC, Canada). The obtained libraries were sequenced in a paired end 150 bp run on Illumina (San Diego, CA, USA) HiSeq 4000 platform.

Raw sequencing reads were quality controlled using FastQC software (Babraham Bioinformatics), and filtered using Trim Galore software (Babraham Bioinformatics) along with adapter sequences removal. Cleaned reads were assembled into cotigs with Shovil v1.1.0 which is an ultra-fast implantation of Spades v3.14.0 algorithm^47^. The obtained contigs were finally polished using Pilon 1.23 software^48^ and scaffolded using MeDuSa web server^49^ against *Paenibacillus lautus* strain E7593-69 (GCA_003590055.1; ASM359005v1) genome. Scaffold belonging to plasmid was identified by Nucleotide BLAST against the reference genome sequence. Contigs statistics were retrieved using Quast v5.0.2 software^50^. The sequences were annotated during genome submission by the NCBI Prokaryotic Genome Annotation Pipeline (PGAP)^51^ using Best-placed reference protein set (GeneMarkS-2+) v5.1. Taxonomic analysis of the isolated strain and search for the most similar spices were done using TYGS web server^52^. Both 16S rDNA (SSU) and whole-genome based phylogenic trees were constructed.

### Preparation of bacterial material for alkaloid and growth regulator analysis

A single colony of pure bacteria cultures was picked up with a pipette tip and inoculated with 50 mL of liquid LB medium in Erlenmeyer flasks (in 50 replicates). The liquid cultures were incubated for 24 h at 37 °C on a shaker at 100 rpm (Innova^®^ 42/42R New Brunswick™ incubator, Eppendorf, Germany). After this time, the cell suspension was centrifuged and the resulting bacterial cell pellet was lyophilized (FreeZone 6 Liter freeze dryer, Labconco, USA).

### Elicitor preparation

One isolated colony of endophytic bacteria was transferred to 10 mL of LB liquid medium in a glass tube via pipette tip and incubated at 37 °C on a shaker at 100 rpm for 24 h. Bacterial quantification was performed using a spectrophotometer (NanoDrop 2000, Thermo Fisher Scientific, USA) at 600 nm. The bacterial culture was established to an optical density (OD) of 1.0^53,54^. Afterward, the bacteria were autoclaved and then centrifuged (6000 × g, 20 min). The supernatant was removed, and the cell sediment was resuspended in 10 mL of sterile distilled water and used for elicitation according to Lim et al.^55^.

### Elicitor treatments

*L. aestivum* plants 12 months old were obtained from somatic embryos and transferred to a liquid MS medium containing 5 µM of zeatin^39^. The experiment was carried out in the RITA^®^ bioreactor (Vitropic, France) according to the physical conditions described in Ptak et al.^39^ (see Supplementary Fig. S1d). Autoclaved *P. lautus* strain extracts were added to the medium at concentrations of 0% (as control), 0.01%, 0.02%, and 0.04%. The pH of the media was adjusted to 5.8. The experiment was established in three replications. About five grams of plants were placed in each bioreactor vessel, 60 plants for each combination were used. The plants were grown for various periods of time: 7, 14 and 28 days. After these culture periods dry weight (DW) was measured for the plants after lyophylization (FreeZone 6 Liter freeze dryer, Labconco, USA). The samples were also taken from each treatment for phytochemical and biochemical analyses.

### Alkaloids analyses in bacteria and plant samples

The alkaloids were extracted from dried plants and bacterial pellets as previously described by Spina et al.^14^, purified and analysed using GC–MS system QP2010-Shimadzu equipment (Shimadzu, Kyoto, Japan) operating according to Saliba et al.’s method^9^. The identification of the alkaloids was performed by comparing the measured data with those of authentic compounds (galanthamine, lycorine) or with literature data as specified in the text. The alkaloids were quantified using LC–MS equipment constituted by U3000-Dionex apparatus and micrOTOF_Q_™ apparatus (Bruker Daltonics, Bruker, Bremen, Germany). An internal standard calibration method along with a nine-point calibration curve (R^2^ = 0.99) using authentic galanthamine and lycorine were used for quantitative analysis of alkaloids. The analysis for quantification of alkaloids were repeated three times.

### Plant growth regulators analysis in bacteria samples

Ultrahigh-performance liquid chromatography–tandem mass spectrometry (UHPLC-MS/MS) was used for the analysis of plant growth regulators analysis according to the method of Hura et al.^56^. Three lyophilized bacterial samples of about 20 mg were added to a stable isotope-labelled internal standard mixture. Samples were extracted (methanol/H_2_O/formic acid, 15:4:1 (v/v/v)) and evaporated under a nitrogen stream (TurboVap LV, Caliper, Hopkinton, MA, USA). After dissolution in 3% (v/v) methanol in 1 M HCOOH, they were cleaned up on hybrid solid-phase extraction columns (Bond Elut Plexa PCX; Agilent Technologies, Santa Clara, CA, USA). Targeted profiling of plant growth regulators and related compounds was conducted in multiple reaction monitoring mode on an Agilent Infinity 1260 UHPLC system (Agilent Technologies) coupled with a 6410 Triple Quadrupole LC/MS with an electrospray interface ion source (Agilent Technologies). Separation was performed on an Ascentis Express RP-Amide analytical column (2.7 μm, 2.1 × 150 mm; Supelco, Bellefonte, PA, USA) in a linear gradient of H_2_O vs acetonitrile with 0.01% (v/v) HCOOH. Further technical details are shown in Supplementary Table S3. As internal standard [15N4]dihydrozeatin (N15-DZ), [15N4]kinetin (N15K), [2H5]benzoic acid (D-BeA), [2H5]indoleacetic acid (D-IAA), [2H4]salicylic acid (D-SA), [2H2] gibberellin A_1_(D-GA_1_), [2H6]cis, trans-abscisic acid (D-ABA), and [2H5]jasmonic acid (D-JA) (CND Isotopes, Quebec, Canada) were used. The analysis were repeated three times.

### Pigments content and antioxidant enzymes activity analysis

For each analysis, 100 mg of fresh plant tissue was homogenated in 1 mL of 80% ethanol and analyzed on a micro-plate reader using a spectrophotometer (Synergy 2, Bio-Tek, Winooski, VT, USA). Concentrations of photosynthetic pigments (chlorophylls *a*, *b* and carotenoids) were determined using a Lichtenthaler and Wellburn^57^ method, and absorbance (λ) was measured for the samples at 470 nm, 648 nm, and 664 nm, respectively.The activities of the antioxidant enzymes catalase (CAT), peroxidase (POD) and superoxide dismutase (SOD) were measured in 100 mg of fresh plant tissue homogenated at 4 °C with a 0.05 M phosphate buffer (pH 7.0) containing 0.1 mM EDTA. Then the homogenate was centrifuged at 10,000 rpm for 15 min. All enzymes activities were measured spectrophotometrically using micro-plate reader (Synergy 2, Bio-Tek, Winooski, VT, USA). Activity of CAT was estimated at λ = 240 nm by calculating the rate of H_2_O_2_ decomposition according to the method of Aebi^58^. Activity of POD was measured as the amount of oxidation products of 1% p-phenylenediamine in the presence of H_2_O_2_ at λ = 485 nm^59^. Activity of SOD was measured by the cytochrome method of McCord & Fiodovich^60^ at λ = 550 nm and defined as the amount of enzyme necessary for inhibition of cytochrome c in a coupled system with xanthine and xanthine oxidase. The enzymatic activity was converted into the amount of protein present in plant tissue according to the method of Bradford^61^. The analysis were repeated three times.

### Scanning electron microscopy

Plants were fixed and scanned with a scanning electron microscope (Jeol, JSM 5410, Japan) according to Ptak et al.^62^.

### Data analysis

The results are expressed as mean values and standard deviation (SD). Statistical analysis of the experiment data was done with analysis of variance (ANOVA). Differences between the means were performed using Duncan’s multiple range test at *P* < 0.05.

## Supplementary Information

Supplementary Information.

## Data Availability

The datasets generated during the current study are available from the corresponding author on reasonable request. The genome-wide shotgun design of the endophytic bacterium has been deposited with DDBJ/ENA/GenBank as part of accession JAIFIS00000000. The version described in this paper is version JAIFIS010000000. The raw sequence data are available [https://www.ncbi.nlm.nih.gov/search/all/?term=JAIFIS010000000].
