# Supplementary material for: Endophytic bacteria from in vitro culture of Leucojum aestivum L. a new source of galanthamine and elicitor of alkaloid biosynthesis
Source: Sci Rep. 2022 Aug 11;12:13700. doi: 10.1038/s41598-022-17992-5 (PMC9371375; doi:10.1038/s41598-022-17992-5)
Supplement: Supplementary file 1 — Supplementary Information. [file 41598_2022_17992_MOESM1_ESM.pdf]

# Supplementary materials for:

# Endophytic bacteria from in vitro culture of *Leucojum aestivum* L. a new source of galanthamine and elicitor of alkaloid biosynthesis

**Agata Ptak[1*], Emilia Morańska[1], Marzena Warchoł[2], Artur Gurgul[3], Edyta Skrzypek[2], Michał Dziurka[2], Dominique Laurain-Mattar[4], Rosella Spina[4], Anita Jaglarz[5] & Magdalena Simlat[1]**

[1]Department of Plant Breeding, Physiology and Seed Science, University of Agriculture in Krakow, Łobzowska 24, 31-140 Krakow, Poland
[2]The Franciszek Górski Institute of Plant Physiology, Polish Academy of Sciences, Niezapominajek 21, 30-239 Krakow, Poland
[3]Centre for Experimental and Innovative Medicine, University of Agriculture in Krakow, Rędzina 1C, 30-248, Krakow, Poland
[4] Université de Lorraine, INRAE, LAE, 54000 Nancy, France
[5]Moredun Research Institute, Pentland Science Park, Bush Loan, Penicuik, EH26 0PZ, United Kingdom

*Correspondence: mfptak@cyf-kr.edu.pl (A. Ptak)

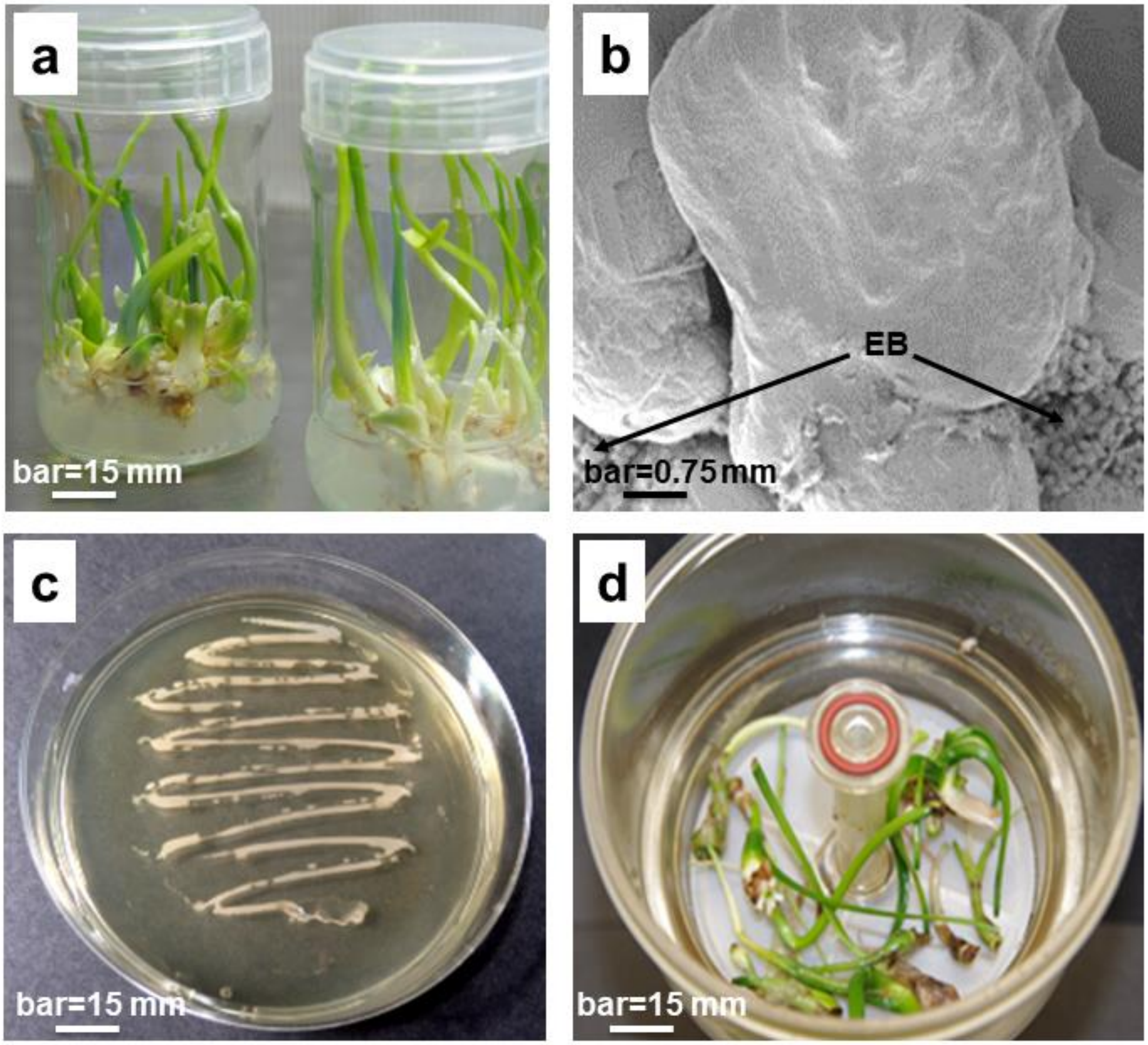


**Figure S1.** *Paenibacillus lautus* strain PLV in *Leucojum aestivum* in vitro cultures: **(a)** micropropagation of *L. aestivum* plants **(b)** scanning electron microscopy of plants showing the colonization by endophytic bacteria (EB) **(c)** endophytic bacteria growth on lysogeny broth medium **(d)** elicitation experiment in bioreactor Rita®.

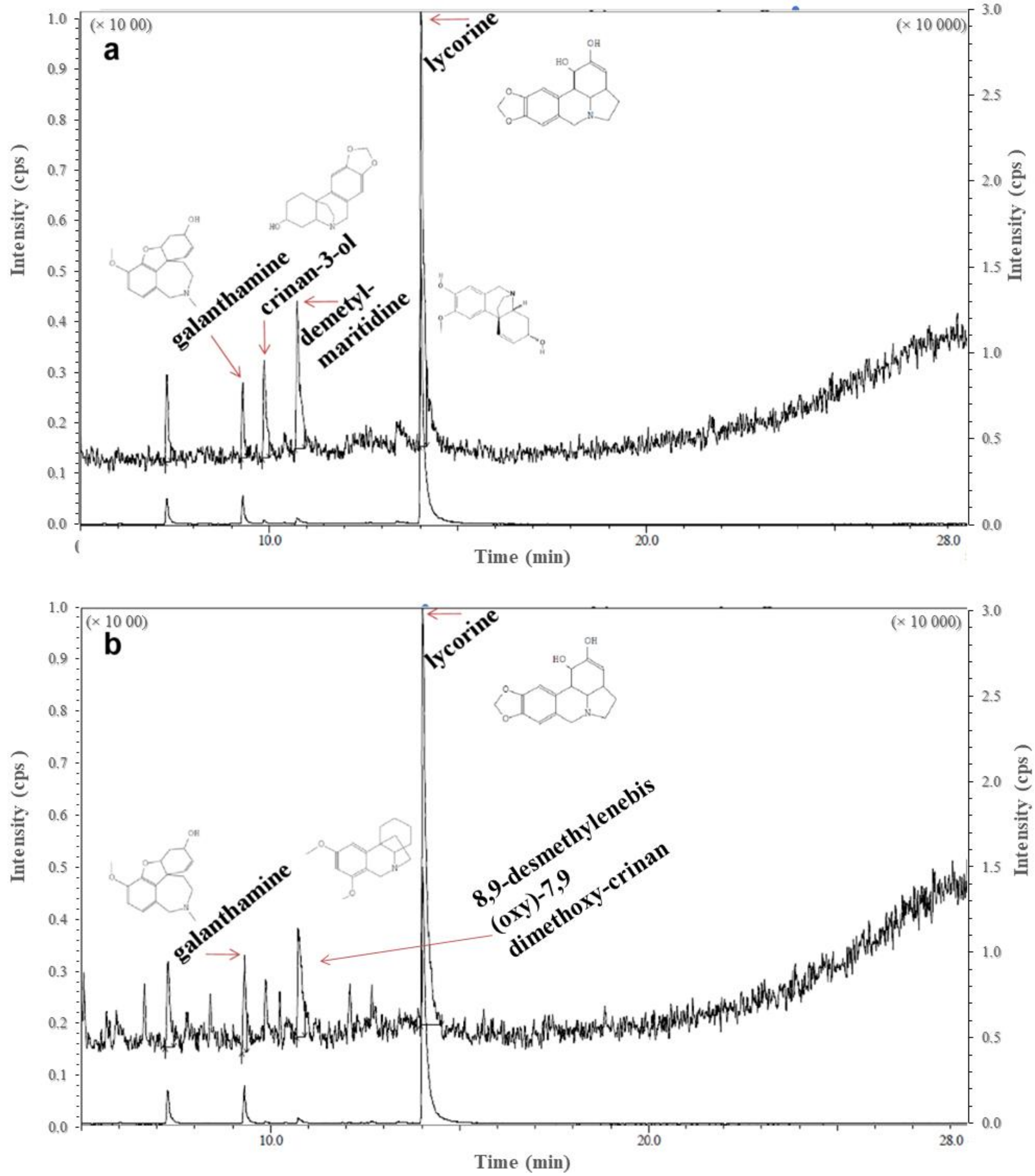


**Figure S2.** GC-MS total ion current chromatograms of the alkaloid fraction extract from *in vitro* plants of *L. aestivum* growth during 28 days on the **(a)** control and **(b)** medium contained 0.04% *Paenibacillus lautus* strain PLV autoclaved extract. Each panel presents a chromatogram acquired for standard solution (lower trace, right y-axis scale) and for authentic extract sample (upper trace, left y-axis scale ).

**Table S1**. Quality assessment for *Paenibacillus lautus* PLV strain assembly.

| Statistics* | |
|---|---|
| Total sequence length | 7,809,730 |
| Total ungapped length | 7,804,930 |
| Gaps between scaffolds | 0 |
| Number of scaffolds | 61 |
| Scaffold N50 | 7,405,488 |
| Scaffold L50 | 1 |
| Number of contigs | 109 |
| Contig N50 | 376,288 |
| Contig L50 | 7 |
| GC (%) | 48.79 |
| # N's per 100 kbp | 61.52 |

*- All statistics are based on contigs of size >= 500 bp, unless otherwise noted (e.g., "# contigs (>= 0 bp)" and "Total length (>= 0 bp)" include all contigs)

**Table S2**. Identified by GC-MS Amaryllidaceae alkaloids in *Paenibacillus lautus* strain PLV with library search matches.

| Alkaloid | Formula | Retention time (min) | Base peak (*m/z*) |
|---|---|---|---|
| Ismine | $C_{15}H_{15}NO_3$ | 7.82 | 238.1 |
| Galanthamine | $C_{17}H_{21}NO_3$ | 9.36 | 286.2 |
| Lycoramine | $C_{17}H_{23}NO_3$ | 9.63 | 288.2 |

Ismine

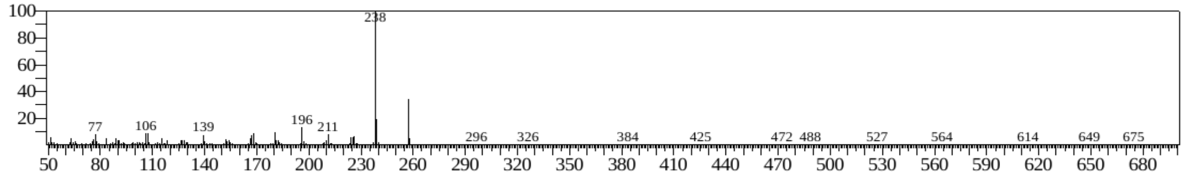


Hit#:1 Entry:11 Library:GALANTHAMINE.lib
SI:80 Formula:C15H15NO3 CAS:0-00-0 MolWeight:257 RetIndex:20
CompName:Ismine

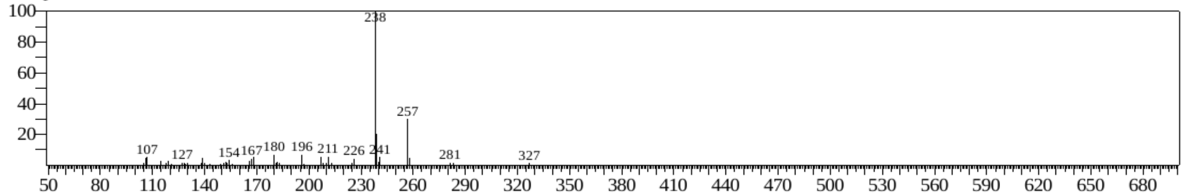


Galanthamine

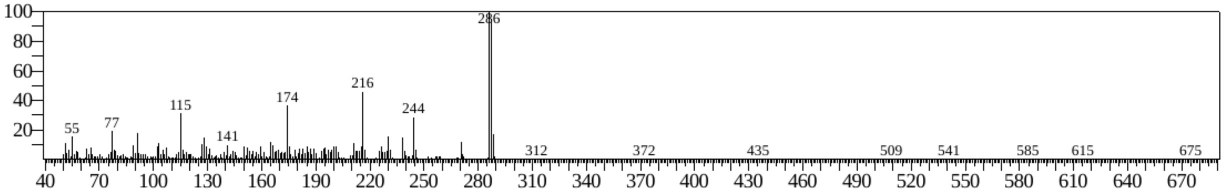


Hit#:1 Entry:102910 Library:NIST08.LIB
SI:88 Formula:C17H21NO3 CAS:357-70-0 MolWeight:287 RetIndex:2213
CompName:Galantamin $$ 6H-Benzofuro(3a,3,2-ef)(2)benzazepin-6-ol, 4a,5,9,10,11,12-hexahydro-3-methoxy-11-methyl-, (4aS-(4a.alpha.,6.beta.,8aR*))- $$ C

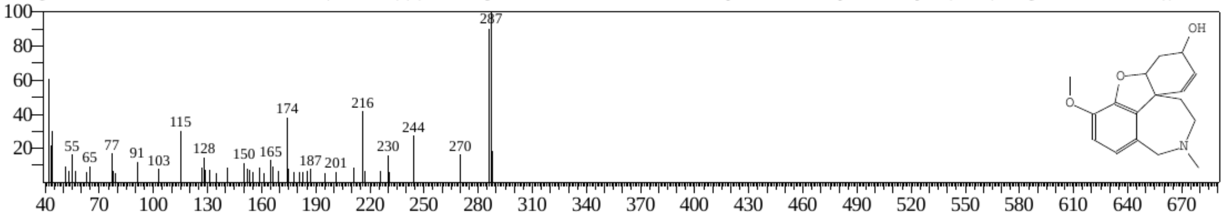


Lycoramine

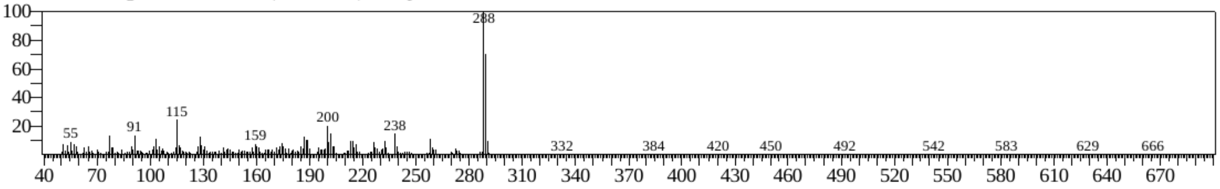


Hit#:1 Entry:104185 Library:NIST08.LIB
SI:72 Formula:C17H23NO3 CAS:21133-52-8 MolWeight:289 RetIndex:2231
CompName:Lycoramine $$ 1,2-Dihydrogalanthamine # $$

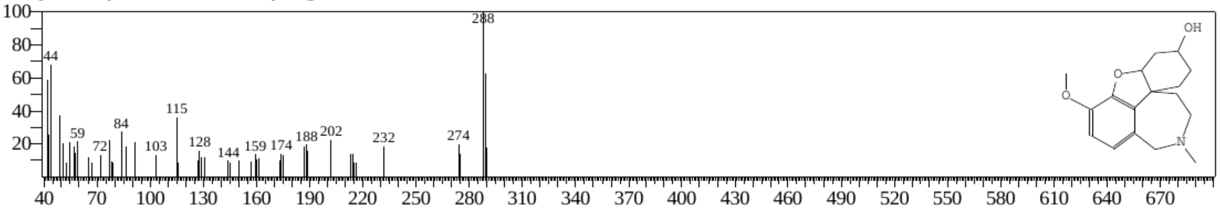

| Alkaloid | Formula | Retention time (min) | Base peak (*m/z)* |
|---|---|---|---|
| Haemanthamine | $C_{17}H_{19}NO_4$ | 12.93 | 272.2 |
| 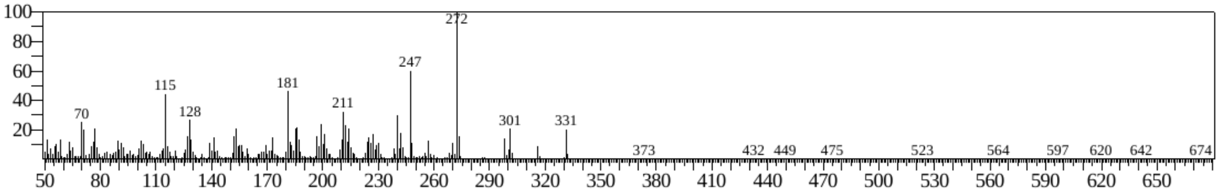  | | | |
| Hit#:1 Entry:9 Library:GALANTHAMINE.lib<br>SI:67 Formula:C17H19NO4 CAS:0-00-0 MolWeight:301 RetIndex:23<br>CompName:Haemanthamine | | | |
| 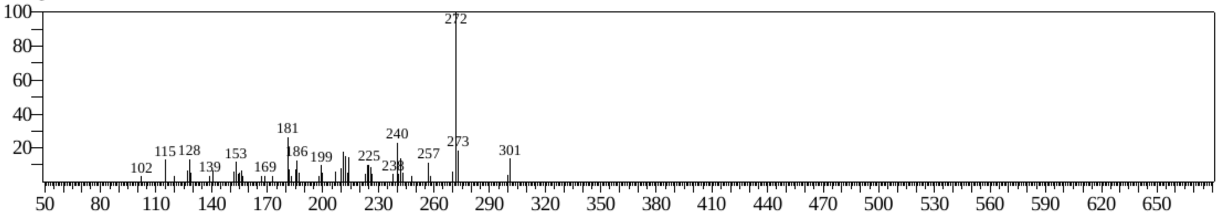  | | | |
| Tazettine | $C_{18}H_{21}NO_5$ | 13.06 | 247.2 |
| 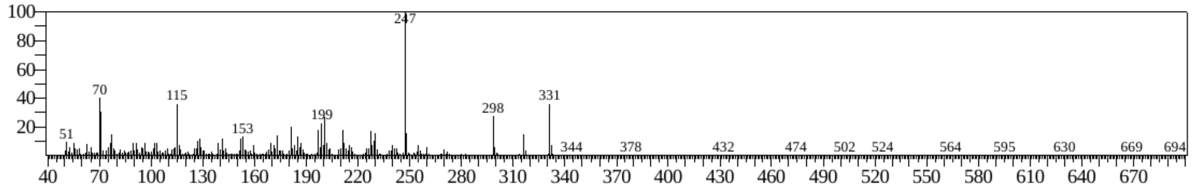  | | | |
| Hit#:1 Entry:25641 Library:NIST08s.LIB<br>SI:86 Formula:C18H21NO5 CAS:507-79-9 MolWeight:331 RetIndex:2415<br>CompName:Tazettine $$ Sekisanin $$ Sekisanoline $$ Sekisanolin $$ Sekisanoline $$ Tazetine $$ Tazettin $$ Ungernin $$ Ungernine $$ 8H-[1,3]Dioxolo[6,7][2]benzopyrano[3 | | | |
| 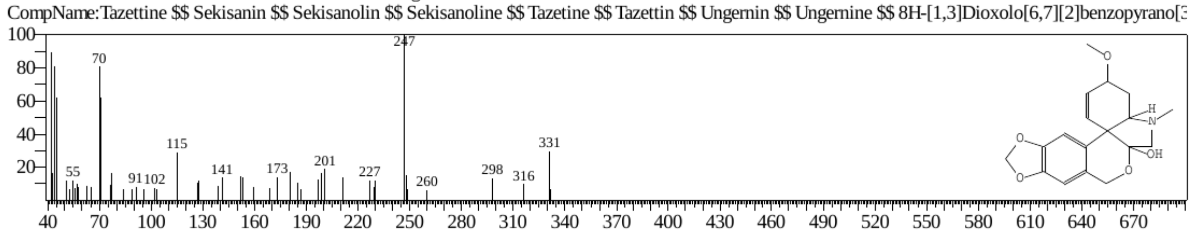  | | | |
| Galanthine | $C_{18}H_{23}NO_4$ | 13.56 | 242.2 |
| 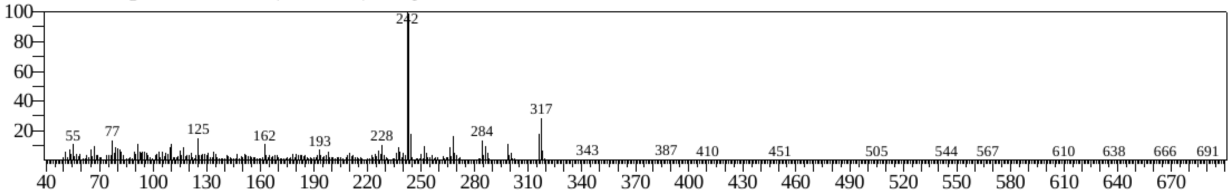  | | | |
| Hit#:1 Entry:25125 Library:NIST08s.LIB<br>SI:87 Formula:C18H23NO4 CAS:517-78-2 MolWeight:317 RetIndex:2333<br>CompName:Galanthan-1-ol, 3,12-didehydro-2,9,10-trimethoxy-, (1.alpha.,2.beta.)- $$ Galanthine $$ 9,10-Secolycoran-1.alpha.-ol, 3,3a-didehydro-2.beta.-meth | | | |
| 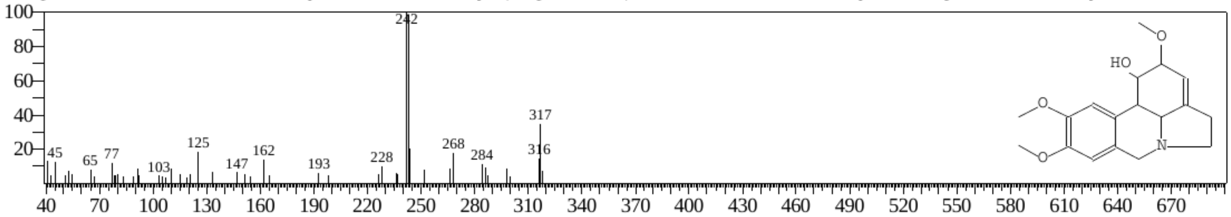  | | | |

| Alkaloid | Formula | Retention time (min) | Base peak (*m/z*) |
|---|---|---|---|
| Lycorine | $C_{16}H_{17}NO_4$ | 14.65 | 226.1 |

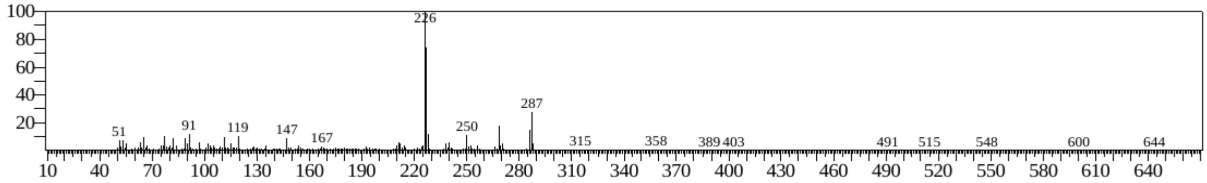


Hit#:1 Entry:102825 Library:NIST08.LIB
SI:89 Formula:C16H17NO4 CAS:0-00-0 MolWeight:287 RetIndex:2263
CompName:Lycorin

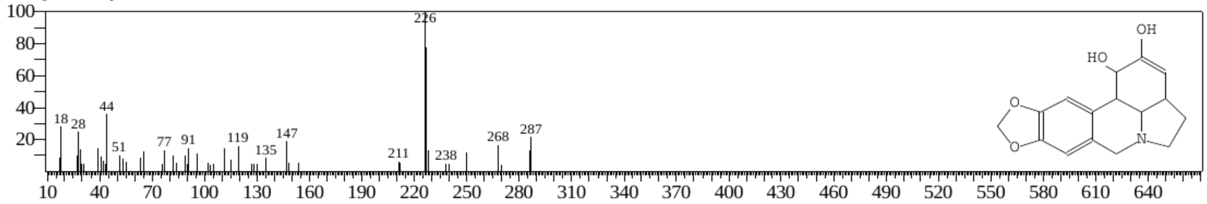


| | | | |
|---|---|---|---|
| Homolycorine | $C_{18}H_{21}NO_4$ | 15.22 | 109.2 |

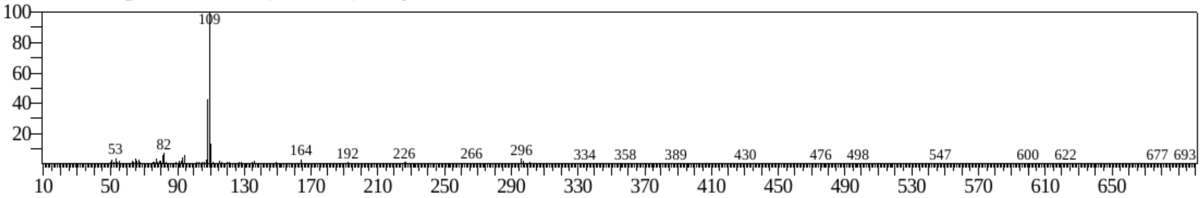


Hit#:1 Entry:122680 Library:NIST08.LIB
SI:88 Formula:C18H21NO4 CAS:477-20-3 MolWeight:315 RetIndex:2415
CompName:Lycorenan-7-one,9,10-dimethoxy-1-methyl- $$ 9,10-Dimethoxy-1-methyllycorenan-7-one # $$

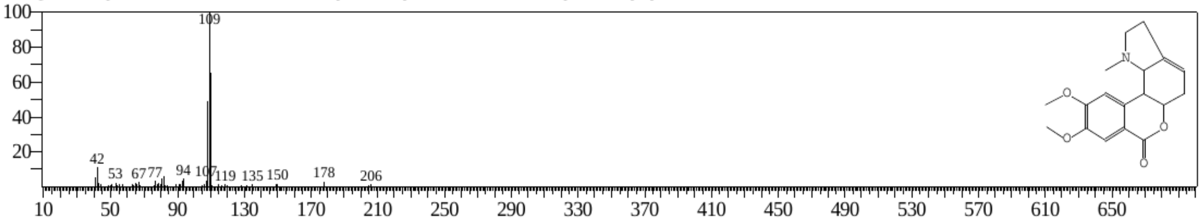


| | | | |
|---|---|---|---|
| 1,2-dihydrochlidanthine | $C_{17}H_{23}NO_3$ | 15.60 | 228.2 |

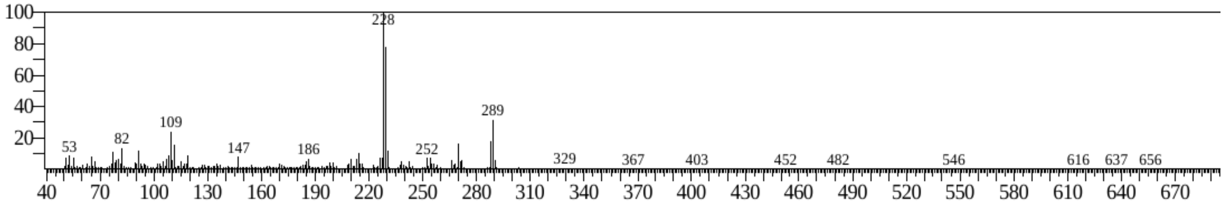


Hit#:1 Entry:104183 Library:NIST08.LIB
SI:85 Formula:C17H23NO3 CAS:111272-18-5 MolWeight:289 RetIndex:2211
CompName:Chlidanthine, 1,2-dihydro- $$ 6-Methoxy-11-methyl-5,6,7,8,9,10,11,12-octahydro-4ah-[1]benzofuro[3a,3,2-ef][2]benzazepin-3-ol # $$

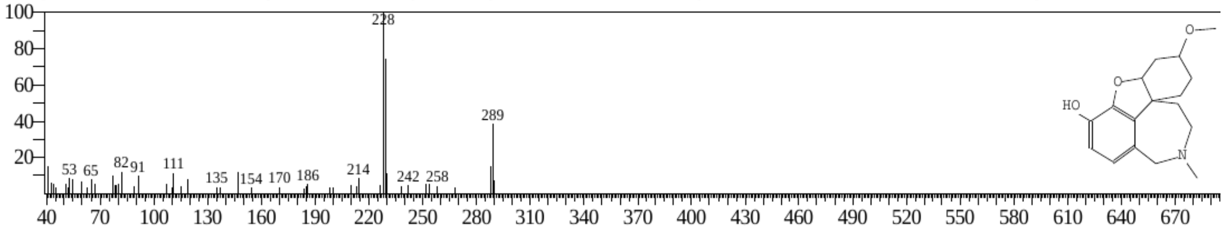

| Alkaloid | Formula | Retention time (min) | Base peak (*m/z)* |
|---|---|---|---|
| Hippeastrine | $C_{17}H_{17}NO_5$ | 16.08 | 125.1 |

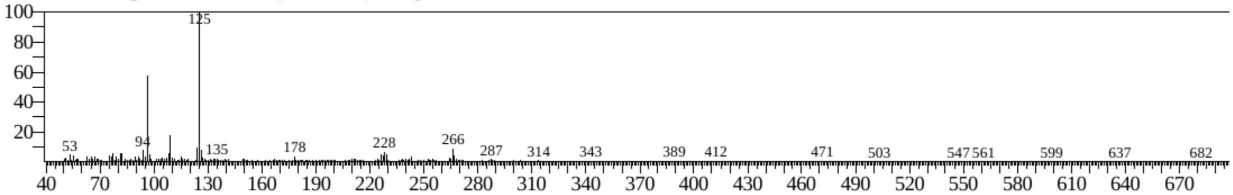


Hit#:1 Entry:25042 Library:NIST08s.LIB
SI:79 Formula:C17H17NO5 CAS:477-17-8 MolWeight:315 RetIndex:2569
CompName:Lycorenan-7-one, 5-hydroxy-1-methyl-9,10-[methylenebis(oxy)]-, (5.alpha.)- $$ Hippeastrine $$ Lycorenan-9-one, 6-hydroxy-1-methyl-12,13-[met

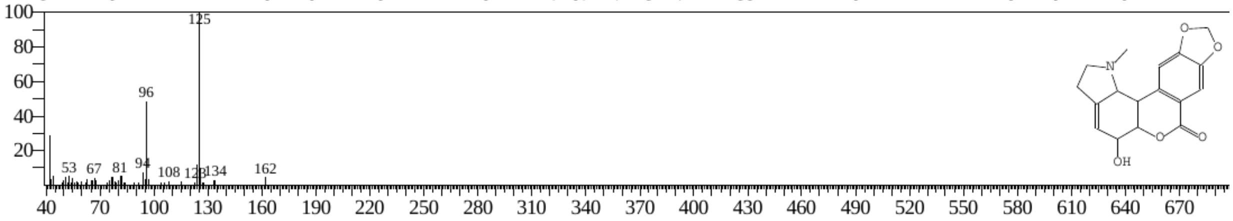

**Table S3**. MRM parameters at positive ion mode (+ESI), capillary voltage 4 kV, gas temperature 350°C, gas flow 12 l/min and nebulizer pressure 35 psi. MassHunter software was used to control the LC–MS/MS system and in data analysis. For MRM parameters optimization MassHunter Optimizer was used.

| Compound* | | Type of ion | Quantifier transition [precursor/product ions] | Fragmentor voltage [V] | Collision energy [V] | Retention time [min] |
|---|---|---|---|---|---|---|
| tZ | | [M+H]+ | 220.2/136.3 | 85 | 9 | 1.95 |
| cZ | | [M+H]+ | 220.2/136.3 | 85 | 9 | 2.75 |
| N15-DZ | ISTD | [M+H]+ | 226.2/140.0 | 124 | 18 | 2.32 |
| K | | [M+H]+ | 216.1/188.3 | 90 | 9 | 4.86 |
| N15-K | ISTD | [M+H]+ | 220.1/192.3 | 90 | 9 | 4.80 |
| BeA | | [M+H]+ | 124.1/80.0 | 60 | 14 | 7.85 |
| D-BeA | ISTD | [M+H]+ | 128.1/84.2 | 60 | 14 | 7.48 |
| IAA | | [M+H]+ | 176.1/130.3 | 51 | 9 | 9.31 |
| D-IAA-D5 | ISTD | [M+H]+ | 181.1/135.1 | 38 | 14 | 9.03 |
| SA | | [M+H]+ | 139.2/121.2 | 80 | 14 | 9.29 |
| D-SA | ISTD | [M+H]+ | 143.2/125.2 | 80 | 14 | 9.01 |
| $GA_1$ | | $[M-H_2O+H]+$ | 331.3/285.3 | 100 | 14 | 10.21 |
| D-$GA_1$ | ISTD | $[M-H_2O+H]+$ | 333.3/287.2 | 58 | 9 | 10.08 |
| ABA | | $[M-H_2O+H]+$ | 247.4/187.2 | 80 | 14 | 17.07 |
| D-ABA | ISTD | $[M-H_2O+H]+$ | 253.4/191.3 | 80 | 14 | 16.89 |
| JA | | [M+H]+ | 211.3/151.2 | 80 | 14 | 18.70 |
| D-JA | ISTD | [M+H]+ | 216.3/153.2 | 80 | 5 | 18.41 |

* zeatin: Z, kinetin: K, benzoic acid: BeA, indoleacetic acid: IAA, salicylic acid: SA, gibberellin $A_1$: $GA_1$, abscisic acid: ABA, and jasmonic acid: JA, ISTD: Internal Standard.
